# Artificial Blood: The History and Current Perspectives of Blood Substitutes

**DOI:** 10.15190/d.2020.1

**Published:** 2020-03-18

**Authors:** Fahad Khan, Kunwar Singh, Mark T. Friedman

**Affiliations:** Mount Sinai Health System, Department of Pathology and Laboratory Medicine, Icahn School of Medicine, New York, NY, USA

**Keywords:** Artificial blood, bloodless medicine and surgery, blood substitute, hemoglobin-based oxygen carrier, PEGylated bovine carboxyhemoglobin, oxygen therapeutic agent, perfluorocarbon.

## Abstract

Blood transfusions are one of the most common procedures performed in hospitalized patients. Yet, despite all of the measures taken to ensure the safety of the blood supply, there are known risks associated with transfusions, including infectious and noninfectious complications. Meanwhile, issues with blood product availability, the need for compatibility testing, and the storage and transport requirements of blood products, have presented challenges for the administration of blood transfusions. Additionally, there are individuals who do not accept blood transfusions (e.g., Jehovah’s Witnesses). Therefore, there is a need to develop alternative agents that can reliably and safely replace blood. However, although there have been many attempts to develop blood substitutes over the years, there are currently no such products available that have been approved by the United States Food and Drug Administration (FDA). However, a more-recently developed hemoglobin-based oxygen carrier has shown promise in early clinical trials and has achieved the status of “Orphan Drug” under the FDA.

**SUMMARY**

1. Introduction
2. Blood vs. blood substitute: not one and the same
3. Perfluorocarbon-based blood substitutes
4. Hemoglobin-based blood substitutes
5. PEGylated hemoglobins
6. Conclusion **

## 1. Introduction

Although the search to discover a safe and effective blood substitute, commonly referred to as “artificial blood” but more scientifically termed “oxygen therapeutic agent” (for reasons described later in this article), has been ongoing for a number of decades, true success has yet to be realized. Blood transfusion is still one of the most commonly performed procedures in hospitalized patients nowadays, and the safety of blood products has increased for several reasons: 1. enhanced health and risk-factor screening of prospective blood donors, 2. increased donor testing for transmissible diseases such as human immunodeficiency virus (HIV) and viral hepatitis (hepatitis B and C viruses) but now also including testing for more-recently recognized transmissible disease risks (for example Zika virus and in some cases, babesiosis, a parasitic disease usually transmitted via tick bite that may be transmitted through blood transfusion), and 3. improvements in the processing of blood products (for example, universal leukoreduction, a process in which contaminating and potentially-harmful white blood cells are filtered out from the blood product)^1-3^. Nevertheless, the risks of blood transfusion cannot be entirely eliminated. Moreover, some recognized risks have proven to be quite challenging to prevent or mitigate, such as iron overload (a result of receiving repeated transfusions of red blood cells [RBCs] which contain 200-250 mg of iron) and transfusion-related acute lung injury (TRALI), a complication in which acute lung swelling (pulmonary edema) classically occurs as a result of antibodies to donor human leukocyte antigen (HLA) in a susceptible patient though alternative pathways have also been described (such as infusion of bioactive response modifiers, or BRMs, that accumulate during storage of cellular blood products [i.e., RBCs and platelets])^4,5^. Meanwhile, there are well-known transmissible disease risks that are not or cannot be tested for; these include malaria transmission, transmission of human granulocytic anaplasmosis (formerly erlichiosis) bacteria, and transmission of prion disease (i.e., variant Creutzfeldt- Jakob disease [vCJD], also known as bovine spongiform encephalopathy, the causative agent of mad cow disease), to name just a few of these risks^6-8^. Emerging, yet to be recognized, risks are also a major concern of blood transfusion.

Availability of blood products and compatibility present additional challenges in the practice of transfusion. Seasonal blood shortages, particularly during the height of the summer and winter holidays, are not uncommonly encountered throughout regions in the United States, sometimes causing elective surgeries to be postponed. Furthermore, there can be great difficulty in finding available blood for patients who are highly immunized (i.e., they have formed many antibodies to minor RBC antigens, a common problem among patients with sickle cell anemia, for example) or for those who have a rare blood type (such as Bombay type, also known as O_h_, present in less than 1% of the world’s population).

Challenges in the management of anemic or bleeding patients are also presented by those individuals who conscientiously refuse blood transfusion on the grounds of religious beliefs (e.g., Jehovah’s Witnesses) or other reasons (i.e., bloodless medicine and surgery patients). For this and all of the above reasons, development of a universal blood substitute would seem like the ideal solution. Nevertheless, the development of such a product has proven to be quite elusive despite many attempts to overcome the barriers to success. This article will further discuss these attempts, the products that were developed and tested, and the failures that occurred. Finally, it will highlight promising newer blood substitutes that are currently undergoing testing, known as PEGylated hemoglobins. It should be emphasized that although the authors attempted to comprehensively cover all known developed products, it is highly likely that some products are not discussed in this article.

## 2**. Blood versus blood substitutes: not one and the same**

Blood, often equated to the substance of life, is involved in a number of important physiologic functions owing to its multiple plasma components and cells. Naturally, blood is central to providing tissues with oxygen and nutrients as well as waste removal. However, circulating white blood cells (granulocytes and lymphocytes [B- and T-cells]) carry out important immune functions while platelets, plasma coagulation and fibrinolysis factors are necessary for the balance of blood clot formation and degradation. Hormone transport is yet another important function of blood. Perhaps less well known is that blood also plays a major role in pH buffering, since the blood pH must be tightly regulated around pH 7.40^9^.

Blood substitutes, on the other hand, are not really a complete substitute for blood as their name would imply, considering that such agents are merely designed to support just one therapeutic function of blood, namely, oxygen transport to the tissues. For this reason, blood substitutes are more appropriately termed “oxygen therapeutic agents” (OTAs)^1^.

## 3**. Perfluorocarbon-based blood substitutes**

### *3.1 Description*

**Figure 1:**
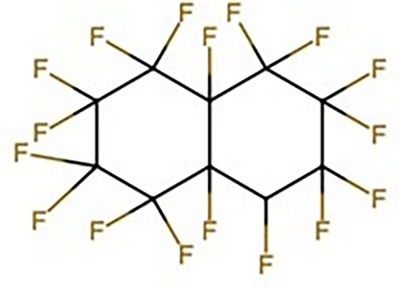
Perfluorocarbon-Based Oxygen Carrier Structure

The ability for perfluorocarbons (PFCs) to be used as oxygen carrying agents was first described by Clark and Gollan in 1966^10^. Their study was able to demonstrate that mice can survive when immersed in an oxygenated PFC solution. PFCs are a synthetic molecule composed of carbon and fluorine atoms. The interaction of these atoms forms a long strong bond that protects it from chemical degradation with fluorine ions forming an electronegative shield around the molecule as seen in **Figure 1**. Due to the hydrophobic qualities, a complex procedure was created to stabilize them in emulsions for intravenous use. When made into this emulsion, PFCs are able to dissolve gases better than most liquids. This is due to fluorine’s low polarizability which decreases the van der Waals interactions between the PFC molecules. These interactions are known to keep together nonpolar molecules. PFCs, in turn, have strong intramolecular bonds making them stable, but they have very weak intermolecular bonds allowing them to behave as gas-like fluids which easily dissolve other substances of low cohesivity (i.e. oxygen [O_2_], carbon dioxide [CO_2_], etc.). The rate of uptake and release of O_2_ by PFCs is unaffected by temperature and environment. In contrast to hemoglobin, which relies on localized chemical bonds for the dissolution of O_2_, PFCs follow Henry’s law that dissolved O_2 _concentration at equilibrium at a given temperature is directly proportional to the gas’ partial pressure, resulting in the rapid and extensive extraction of O_2_ when needed. The practicality of PFCs being a good candidate for in vivo use comes from its inert qualities to dissolve gases, its combination of O_2_/CO_2_ solubilities, and its molecular stability.

The exceptionally strong intramolecular binding and low intermolecular cohesiveness of liquid PFCs result in unique properties that make them different from other regular organic compounds. Fluorinated compounds can reach a level of effectiveness in performance that cannot be achieved by non-fluorinated compounds. In summary, fluorinated compounds such as PFCs offer unique combinations of properties that can make them irreplaceable and constitute the basis for potential biomedical applications^11^.

### ** *3.2 Developed products*

The first generation of PFC emulsions to be developed was Fluosol-DA (Green Cross Corp., Osaka, Japan), which was engineered over 30 years ago (**Table 1**). The chemical composition of Fluosol was a 7 to 3 ratio of perfluorodecalin and perfluorotripropylamine while using pluronic F-68 (a mixture of short- chain linear polymers; Wyandotte Chemicals Corp., Wyandotte, MI) as an emulsifier. It was available as a 20% solution with the ability to carry 7.2% volume of 100% O_2_ at 37°C. This compound also could carry 34% of the O_2_ content of whole blood at a hemoglobin level of 14 g/dl^12^. The first use of this commercially-available product was reported in Japan with infusion of 500 ml and 1000 ml given to patients with severe gastrointestinal bleeding and surgery-related blood loss for esophageal cancer, respectively^13,14^. Subsequent in vivo and in vitro clinical trials after the initial use in Japan examined Fluosol use in a multitude of patients with different clinical issues such as severe anemia, peripheral vascular disease and extensive blood loss from a surgical wound while also being tested in patient population that normally refused blood products (e.g., Jehovah’s Witnesses). These published trials were able to demonstrate that Fluosol, the first widely available PFC emulsion, had its merits with a so called “proof of principle” as an injectable tissue oxygenation agent. Over 20 years, the use of Fluosol in laboratories throughout the world allowed for an extensive review of PFCs and was important in the development of concentrated, room temperature-stable, second-generation PFCs.

**Table 1:** Perfluorcarbon Products

Product	Manufacturer	Location of Clinical Use	FDA Approval Status	Current Status
Flusol-DA-20	Green Cross Corporation (Osaka, Japan)	Japan United States	Yes in 1989	Discontinued due to side effects with limited success
Oxygent	Alliance Pharmaceutical Corporation (San Diego, CA)	Europe China United States	Not approved; reached phase II trials	Discontinued due to costs
Oxycyte	Synthetic Blood International (Costa Mesa, CA)	United States	Not approved; reached phase IIB trials	Discontinued due to lack of enrollment into phase II trials
Perftoran	Russian Academy of Sciences (Puschino, Russia)	Russia Mexico	Not approved	Rebranded as Vidaphor (Fluor02 Therapeutics, Inc., Boca Raton, FL) in the United States and currently awaiting clinical trials

Oxygent^TM^(Alliance Pharmaceutical Corp., San Diego, CA) is a stable second-generation concentrated PFC. The original formulation consisted of 60 grams PFC/dL with two active ingredients: perfluorooctyl bromide and perflubrodec. The latter was added in small quantity to stabilize particle growth during storage^15^. To make this product, an egg-yolk phospholipid was used as an emulsification agent for PFCs in a buffered electrolyte solution. The final product had an approximate 24-month shelf life when stored between 2 - 8^o^C but was stable enough to tolerate exposure at room temperature for a few weeks. Over 250 preclinical studies were performed to understand the safety of Oxygent in various animal species before moving on to studies that demonstrated efficacy in humans. Of note, toxicology studies have shown that the emulsion is well tolerated without severe adverse effects when given at appropriate clinical dosing (1.0 - 6.0 ml/kg). In Europe, a large phase III multicenter study showed that Oxygent in conjunction with hemodilution decreased the need for RBC transfusion in 492 patients undergoing non-cardiac surgery^16^. Patients were randomly distributed into two groups: included in the control group were patients that were transfused intraoperatively at a hemoglobin concentration of less than 8.0 g/d while in the test group, patients first underwent acute normovolemic hemodilution to a hemoglobin of 8.0 g/dL then were dosed with Oxygent. Results showed that 26% of patients in the test group had a reduction in the number of RBC units transfused when compared with the control group (16%). However, in the United States. a second trial was terminated because of possible increase in stroke rates in the test arm^17^.

Oxycyte™ (Synthetic Blood International, Costa Mesa, CA) is similar to Oxygent as an emulsified compound with purified egg yolk phospholipids composed of C10F20 PFCs. This product was initially tested in phase II trials where patients who had traumatic brain injury were given the emulsified product^18^. Unfortunately, at this time there are no published data available from this trial, but the company did claim that the initial goal of increasing the partial pressure of arterial O_2_ levels was met. Again, like Oxygent, a second trial was suspended^19^.

### *3.3 Limitations*

PFCs are ambitious products that in theory and chemically seem to be a viable option for clinical use. However, issues with manufacturing and development have led them to become mostly discontinued. Fluosol, as mentioned above, demonstrated this “proof of principle” which led to a newer formulation being FDA approved in 1989, but it eventually suffered from clinical shortcomings. When put into clinical use, the formulation only delivered 0.4 mL of O_2_ per 100 ml. This in turn required patients to receive supplemental O_2_ because most of the O_2_ carried by the substance was unloaded before reaching the microvasculature. Another issue with the product was that it was excreted slowly with metabolites remaining in the body for months after administration. It also had biological side effects such as a temporary decrease in the platelet count and fevers. Lastly, due to the short stability time of approximately 8 hours, it created difficulties for use in coronary balloon angioplasty, the indication for which the product had gained its approval. This led the FDA to remove Fluosol from the market in 1994.

Second-generation iterations of PFCs also had their pitfalls with O_2_ delivery; patients had to receive supplemental O_2_ inhalation because the delivery was less than 30% of normal blood^18^. Oxygent use in a large multicenter study also showed more serious adverse effects/events in the PFC group vs. the control group (32% vs 21%) with a possible increase in stroke rates seen in the United States phase III trial. Most studies on Oxygent have been terminated because of costs and a suggestion that the manufacturers did not have faith in the product’s clinical or commercial success.

Due to initial clinical trials with these products showing lack of beneficial outcomes, no current trials are ongoing with PFCs. There are certain products that are being used in Mexico and Russia (Perftoran^Ⓡ^, Russian Academy of Sciences, Puschino, Russia), but, unfortunately, until a well-designed trial is performed in the United States, PFCs will not be widely used. Currently, Perftoran (rebranded as Vidophor^TM^ [Fluor0_2_ Therapeutics, Inc., Boca Raton, FL] in the United States), is awaiting clinical trials^20^. Ultimately, due to the lack of clinical evidence and the timeline for approval from the FDA, PFCs are unlikely to be a viable blood substitute any time in the near future.

## 4**. Hemoglobin-based blood substitutes**

### *4.1* *Description*

Efforts to develop alternatives to blood date back to the 17^th^ century, albeit with limited success, and still many efforts are currently underway to find a synthetic substance that can cope with the challenges of a hemoglobin-based carrier that will efficiently deliver O_2_ to the tissues without any of the associated toxicities. Currently, due to discouraging adverse events during phase II and III studies, none of the products that have been developed to date were successful in getting FDA approval for use under clinical settings, though hemoglobin-based oxygen carrying (HBOC) agents have been approved and used clinically in two countries (Russia and South Africa) outside of the United States^21-23^. Yet these endeavors have brought new insights into the basic biophysical characteristics of hemoglobin and oxygen transport systems and have paved the way for the development of products which may be readily available with efficient O_2_-carrying capacity and minimal adverse interactions with biophysical hemodynamics^24,25^. In that manner, hemoglobin-based products have been developed as supplements or pharmaceutical-bridging agents to allogeneic RBC transfusion.

**Figure 2:**
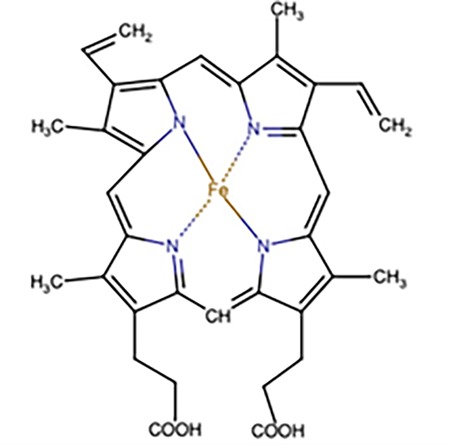
Hemoglobin structure

In simplified terms, hemoglobin (**Figure 2**) is a globular protein having a quaternary structure and consisting of two α and two β chains with four heme groups that are themselves made up of porphyrin rings with a central iron atom that can bond to O_2_. This oxygen-heme bond results in a conformational (shape) change in the hemoglobin molecule which in turn progressively increases the affinity of hemoglobin for additional O_2_ molecules (i.e., O_2_ bonding occurs in a cooperative manner). Thus, a small change in O_2_ partial pressure results in a large change in the amount of O_2_ bound or released by hemoglobin. A number of additional factors, such as temperature and pH, can alter the oxygen-hemoglobin dissociation curve. Similarly, 2,3-diphosphoglycerate (2,3-DPG), a highly-anionic product of the RBC glycolytic pathway and present in normal human erythrocytes (RBCs), also directly affects the binding of O_2_ to hemoglobin. As the concentration of 2,3-DPG rises, the oxygen-hemoglobin dissociation curve “shifts to the right”, thereby allowing the release of O_2_ to tissues at higher than normal O_2_ partial pressures.

**Figure 3:**
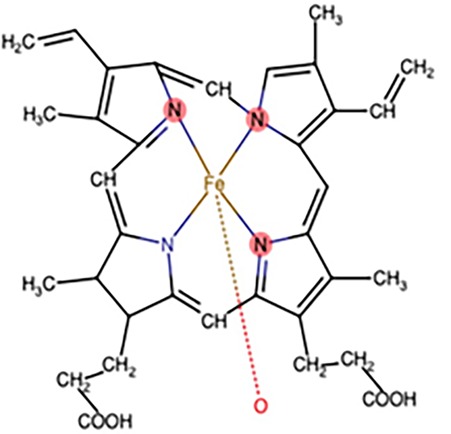
Hemoglobin-Based Oxygen Carrier Structure

Cell-free hemoglobin can potentially be used as a blood substitute because hemoglobin maintains its ability to transport O_2_ outside of the RBC; however, unfortunately, it is quite toxic to surrounding tissues. Toxicity includes endothelial scavenging of nitric oxide (NO) which leads to vasoconstriction (i.e., narrowing of the blood vessels) and the development of O_2_-, heme-, and globin-based radicals^26^. In addition, cell-free hemoglobin in the absence of 2,3-DPG is an extremely inefficient O_2_ transporter, capable of off-loading only a very small amount of O_2_ to the tissues. Owing to this highly-reactive and toxic nature of cell-free hemoglobin as well as the necessary-presence of 2,3-DPG for O_2_ transport efficiency, the development of HBOCs (**Figure 3**) has been quite challenging. Yet, successful development of an HBOC blood substitute is quite advantageous in that compatibility testing (i.e., crossmatching) is not required prior to infusion, it can be sterilized by ultrafiltration and low heat (pasteurization) to inactivate infectious agents, and could have a long shelf life, none of which are characteristic of donor RBC products.

### *4.2 Developed products*

The first generation of blood substitutes to be produced were the stroma-free hemoglobin (SFH) products. SFHs were prepared by lysis of packed RBCs forming soluble hemoglobin. This mixture was then centrifuged to remove the bulk of red cell stroma, leading to the production of SFH. The procedure resulted in 500 cc’s of a 7 g/100 ml solution of hemoglobin with normal physiologic concentration of sodium, potassium, and bicarbonate. The pH of the solution was 7.1 to 7.2, and the osmolality was 270 to 280 mOsm/kg. The methemoglobin concentration ranged from 7 to 12% of the total hemoglobin concentration which did not change significantly over a four-week storage period at 4^o^C^27^. SFH’s could be prepared by either ultrafiltration or crystallization. SFH prepared by ultrafiltration was characterized by a substantially lower content of residual membrane phospholipid and a more restricted protein composition. This preparation was also essentially free of vasoconstrictor and contractility-depressant actions on the ex vivo perfused heart. In contrast, crystallization-produced SFH resulted in a product that was less well purified of both phospholipid and protein constituents. Thus, it was likely to generate denatured protein aggregates during storage and exhibited vasoconstrictor and contractility-depressant activity which could vary significantly from batch to batch. These findings indicated that preparative methodology based on ultrafiltration and size-exclusion yielded SFHs which were superior in respect to those produced by the crystallization method^21^.

The second-generation HBOCs (**Table 2**) were pyridoxilated hemoglobin-polyoxyethylene conju-gates (PHPCs) that have been prepared through chemical modification of SFH. These products have been designed to prevent the major disadvantages of SFHs reported in multiple studies, namely, increased O_2_ affinity, short circulatory half-life, and nephrotoxicity^17^. PHPC is prepared after acquiring SFH; the free hemoglobin is pyridoxilated (i.e., addition of vitamin B6) to adjust the O_2_ affinity and later conjugated with α-carboxymethyl-ω-carboxymethoxy-polyethylene to increase the molecular weight and ensure a longer circulatory half-life. The O_2_ affinity of such crosslinked hemoglobin was found to be higher (i.e., lower P50, the PO_2_ at which hemoglobin is half saturated with O_2_) compared to native human RBCs, and it remains in a deoxy (T) state with the utilization of 2,3-DPG analogs^28^. The second-generation HBOCs that have been developed are PolyHeme^Ⓡ^(Northfield Laboratories, Evanston, IL), Hemopure^Ⓡ^(also referred to as HBOC-201; Hemoglobin Oxygen Therapeutics LLC, Souderton, PA), and HemoLink^TM^(Hemosol Inc, Mississauga, Canada). However, many of these products have been discontinued owing to the observed adverse events in clinical trials^27^. Hemopure, however, is approved by the South African drug council for the treatment of anemia dating back to 2001, and the product is available in the United States for qualified patients (such as Jehovah’s Witnesses who do not accept blood) with life-threatening anemia under the FDA’s expanded (compassionate use) access program^29,30^. In addition, one randomized, multicenter trial investigating the safety and efficacy of HBOC-201 in non-cardiac surgery patients did find that use of the HBOC product reduced RBC transfusions in 43% of patients without notable differences in mortality and serious adverse events though there was a notable excess of non-serious (i.e., hypertension and fever) adverse events associated with HBOC-201^31^.

**Table 2:** Second-Generation Hemoglobin-Based Oxygen Carriers

Product	Manufacturer	Location of Clinical Use	FDA Approval Status	Current Status
PolyHeme	Northfield Laboratories (Evanston, IL)	United States	Not approved; reached phase III clinical trails	Approved in South Africa; currently phase II trials are on hold due to safety issues
Hemopure	Hemoglobin Oxygen Therapeutics LLC (Souderton, PA)	United States Europe	Not approved; available through FDA expanded (compassionate use) access program	FDA expanded access program allows product to be used for qualifying patients with severe, life threatening anemia after exhausting all other options
HemAssist	Baxter International Corporation (Deerfield, IL)	United States	Not approved; reached phase III trials	Discontinued; phase III trials were halted due to low efficacy and safety record
Hemolink	Hemosol Inc. (Mississauga, Canada)	North America	Not approved, reached phase II trials	Trials ongoing in sickle cell disease patients, and in cardiac surgery

The third-generation blood substitutes include hemoglobin crosslinked between the α chains with bis(dibromosalicyl) fumarate (DBBF) or αα-hemoglobin. This product was developed at the Letterman Army Institute of Research (LAIR) in San Francisco, CA. Similarly, Baxter International Corporation (Deerfield, IL) developed HemAssist^Ⓡ^, a diaspirin-crosslinked hemoglobin (DCLHB). These developed products were more homogenous to support the major toxicological and physiological experiments and also have O_2_ affinity similar to that of blood^32^. The products are the result of very-well documented steps presented by the LAIR and Baxter International Corporation^29,30,33,34^. They were made using outdated human or bovine blood, washing the RBCs with sterile saline to remove all the traces of plasma, and then subjecting them to hypertonic lysis. The remaining membrane material was filtered out, allowing for the purified hemoglobin to be put towards the crosslinking reaction and resulting in the purified crosslinked hemoglobin with an overall yield of 55% to 58%^29,35^. To maintain specific crosslinking between lysine 99α residues, an allosteric effector (2,3 DPG pocket) was added to keep the hemoglobin in the deoxygenated (T) state. In the final stage, the reagent DBBF was added; this mixture was then heated to remove unreacted hemoglobin as well as pathogens. The final product was formulated in either Ringer’s lactate or Ringer’s acetate and could be stored frozen at -20^o^C for up to one year^32^. These products, however, lack the ability to regulate the oxidative state caused by iron in their heme group, and their phase III clinical trials have shown increased mortality with their use^29^. Nevertheless, Hemolink™ a noteworthy and relatively newer intermolecular crosslinked hemoglobin with activated sugar, O-raffinose combination, has been more recently developed by Hemosol Inc. The biophysical properties include oxygen-carrying characteristics (P50 of 30–40 mmHg) with non-cooperative behavior (Hill coefficient (n) = 1, compared to 2.5–2.9 for normal human hemoglobin). The product has been tested in phase I and phase II clinical trials in patients undergoing elective coronary artery bypass graft surgery, (CABG), yet no phase III trial data is available publicly^36-38^.

### *4.3 Limitations*

As mentioned above, several serious adverse events have resulted in premature termination of HBOC clinical trials. The following is a succinct description of these key limitations. Vasoconstriction leading to tissue hypo-oxygenation and subsequent systemic hypertension and pulmonary hypertension is the most feared limitation of HBOCs. Studies have shown that NO-scavenging by free hemoglobin has been the major factor mediating vasoconstriction^36^. NO regulates endothelial mechanisms of smooth muscle relaxation by limiting the production of a vasoconstrictor known as endothelin. HBOCs are carried in the plasma and therefore free to cross through the endothelium, thereby this free hemoglobin has the potential to consume larger amounts of NO to regulate the vascular smooth muscle tone^37^. Other proposed mechanisms of HBOC-induced vasoconstriction include the release of norepinephrine from adrenergic regulation of the peripheral nerves and neurally-mediated distortion of chronotropic and inotropic myocardial responses as well as the disturbed neural response in maintaining smooth muscle tone^39^. Vasoconstriction to a certain extent has been noted to be less severe in polymerized hemoglobin preparations^39-41^. The phenomenon of vasoactivity and dysregulated smooth muscle tone may manifest clinically in the form of gastrointestinal distress, flu-like symptoms, and nephrotoxicity^42,43^.

Compared to RBCs, HBOCs lack the remarkable free-radical scavenging system which includes enzymes superoxide dismutase and hemoglobin reductase. Moreover, free hemoglobin can participate in a number of reactions that could potentially produce toxic free radicals^44^. The iron in free hemoglobin can oxidize to form methemoglobin (HFe^3+^) which reacts with NO to disturb the regulation of smooth muscle tone. Also, HBOCs can induce dysregulation of other physiologic functions at the vascular endothelium by extracellular hemoglobin. Free hemoglobin gets oxidized and produces unneutralized H_2_O_2_ which oxidizes ferrous and ferric hemoglobins to Fe (IV)-ferryl hemoglobin and oxyferryl hemoglobin, respectively. Ferryl hemoglobin can react with H_2_O_2_, yielding free iron and other heme degradation products. Fe (III) hemoglobin produced during hemoglobin autoxidation also readily releases heme, an additional source for oxidative stress and oxidative reactions in the plasma. The proinflammatory effects of heme and oxyferryl have been shown to also increase the risk of further oxidative stress^45^. Methemoglobin is also the degradation product of hemoglobin, production of which is physiologically held in control by the enzyme methemoglobin reductase in the erythrocyte. Therefore, HBOCs have the propensity of producing highly-oxidative free radicals, capable of inducing cell damage and halting other biochemically essential processes^46^.Substances do not normally cross the intact blood-brain barrier unless there is injury or a diseased state in which the sanctity of the blood-brain barrier is compromised. For example, in the case of head injury, cerebral hemorrhage, or ischemic injury to the brain, free hemoglobin in the HBOCs then may cross the barrier and act as a potential neuro-excitatory toxin. In addition, HBOCs may cause reperfusion injury as a result of their high O_2_ content^47^.

## 5**. PEGylated hemoglobin**

### *5.1 Description*

The biochemical and physiological drawbacks of previously studied generations of OTAs have paved the way for the development of a substance that can bypass the toxic chemical and biophysical interactions of the synthetically-derived blood products, including the vascular tone and oxidative cellular damage. The proposed mechanisms of such toxic interaction have been described above and elsewhere in detail. Based on the preclinical and clinical studies, researchers are able to formulate hemoglobin molecules which are physiologically designed and tuned in such a way to exert minimal vasoactive effects to the microcirculation^48,49^. In addition, their essential features are increased O_2_ affinity (reduced P50), minimal NO scavenging, and increased molecular size and chemical homogeneity^50^.

### *5.2 Developed Products*

**Figure 4:**
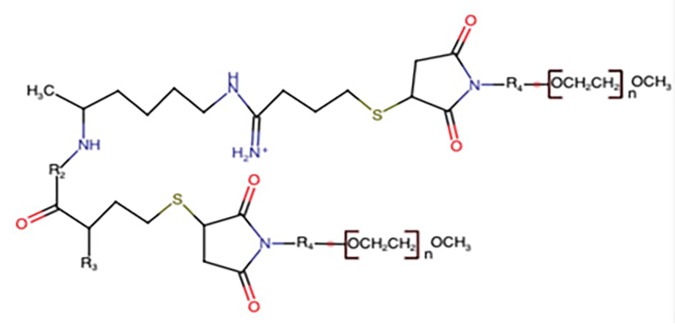
PEGylated Hemoglobin-Based Oxygen Carrier Structure

PEGylated (i.e., addition of polyethylene glycol, **Figure 4**) hemoglobin derived from either human or bovine sources is further modified with maleimide (Hemospan^Ⓡ^, Sangart, San Diego, CA) or carboxylated (Sanguinate^Ⓡ^, Prolong Pharma-ceuticals, South Plainfield, NJ) to produce a hemoglobin with unique O_2_ transport functions (**Table 3**). After multiple failed clinical trials of earlier generation HBOCs, it was clearly evident that these products are not clinically or biochemically identical to human hemoglobin, and due to this major drawback, it was difficult to get regulatory approval for additional large-scale human studies. With these insights, the research community shifted its focus towards developing an O_2_-bridging agent which can be utilized in situations where either blood is not available or where there are other limitations or contraindications for RBC transfusion. In particular, these bridging agents may be advantageous to prevent or treat ischemia-related morbidity and to potentially reduce mortality and thereby demonstrate compelling efficacy to satisfy the regulatory requirements for further studies in acute clinical settings. As a result, usage of the term HBOC was discouraged in the medical literature and the new term, “oxygen therapeutic agent” (OTA), was coined^51^.

**Table 3:** PEGylated Hemoglobin Oxygen Carriers

Product Name	Manufacturer	Location of Clinical Use	FDA Approval Status	Current status
Hemospan	Sangart (San Diego, CA)	United States Europe	Not approved; reached phase III trials in Sweden and phase II trials in the United States	Last documented clinical trials in phase II in Australia and Brazil in 2013; not in clinical use as of 2019
Sanguinate	Prolong Pharmaceuticals (South Plainfield, NJ)	United States	Orphan drug designation from FDA	Used in patients with vaso-occlusive crisis of sickle cell disease

Hemospan, also referred to as MP4, is a human hemoglobin-based agent that was developed to mediate the vasoactivity and hypertensive effects of previously developed PEGylated hemoglobin molecules. Acharya and colleagues have thereby developed an intermolecularly crosslinked hemoglobin with surface conjugation of maleimide-activated 5 kDa PEG to surface thiol groups^52^. The final physical properties of the product differ from that of human blood in that it has a molecular weight of 95 kDa and is more hyperoncotic with slightly lower viscosity relative to blood. Moreover, the P50 is substantially less than blood (6 vs. 28 mmHg at 37^o^C) and has reduced cooperativity (Hill coefficient) and Bohr Effect (pH- dependent O_2_ binding) properties, the reason for which is not well understood. These physical properties render MP4 more homogenous while the molecular design prevents its dissociation into tetramer-dimer fractions which ultimately renders it free of inducing renal tubular damage. In addition, with its increased oncotic pressure, the vascular volume remains preserved. As a general rule, the larger molecules tend to be less vasoactive, and the larger size of MP4 keeps the hemoglobin molecule in the plasma where it has decreased propensity of NO scavenging comparatively to the hemoglobin in the interstitium, which is another means of limiting vasoactivity and hypertension. Based on the study results of Vandegriff and colleagues, the autoregulatory vasoconstriction induced by cell-free hemoglobin is secondary to its high P50 and increased diffusion^50^. The unique molecular design of MP4 allows for hemoglobin O_2_ to be maintained in its low P50 state that facilitates uniform diffusion of hemoglobin along its saturation gradient in the vascular channels with ultimate offloading of O_2_ taking place in the capillaries, which is dependent on the concentration gradient between the capillaries and the perfused tissue. Vandegriff et al. predicted MP4 to be most beneficial in situations where end organ tissue PO_2_ is low and with limited perfusion such as in the states of shock or traumatic ischemia^50^. The clinical efficacy and safety profile of MP4 was assessed in a multicenter study which supported the promising biochemistry of the molecule. The study did not report any clinically-significant adverse events with less frequent hypotensive episodes, though a transient rise in liver enzymes was noted, in patients undergoing major orthopedic surgery^42^. The study posited that MP4 may be of most use in clinical settings where swift O_2_ delivery to the ischemic tissues is necessary^48,53^. Several phase II studies in the United States and phase III studies in Europe were undertaken; however, the study data regarding the efficacy and clinical safety of the product has not been reported and to date, no official statement from the manufacturer has been publicized^54^.

Sanguinate is a PEGylated and carbon monoxide-hybridized (PEG-COHb) OTA with modes of action that may be helpful to mitigate the limitations of the earlier generations of HBOCs. This novel OTA is currently being extensively studied for its characteristics vasodilatory and non-inflammatory properties including the potential to induce minimal to negligible reperfusion cellular injury^45^. PEG-COHb has several modes of actions rendering its multifaceted therapeutic utility beyond anemia into indications that include early brain injury and delayed kidney graft function, where inflammation plays a pivotal pathological role, as well as in indications such as sickle cell disease where the inflammation and hypoxia contribute to the disease progression and associated comorbidities such as vaso-occlusive crisis^55-58^. The design characteristic of PEG-COHb is a modified bovine-source hemoglobin which is polymerized and carboxylated with a potential of rapidly releasing CO and increasing levels of whole blood carboxylated hemoglobin stores to 5.3 to 4.9% in in vitro rat models. This enables O_2_ binding to the CO sites and ultimate end organ tissue oxygenation under hypoxic conditions with the average P50 of 5 mmHG^59^.

Carboxylation of endogenous hemoglobin through the enzyme hem-oxygenase had proven to be of clinical value where oxidative and immune mediated tissue damage is feared. Similarly, synthetically derived CO-releasing molecules have the potential to be more cytoprotective and pleiotropic with remarkable oxidative stress-reducing properties. Studies have demonstrated the anti-inflammatory properties of PEG-COHb with a decrease in inflammatory cytokine levels in patients with acute subarachnoid hemorrhage and in patients with acute sickle cell crisis^54^. The in vitro results suggest PEG-COHb has the potential to improve sickle cell morphology by reverse sickling, and current phase II trials are currently underway examining the value of PEG-COHb in sickle cell vaso-occlusive crisis, acute chest syndrome, and hyperhemolysis where indexed patients are treated with PEG-COHb under compassionate use. Also, there are reports of PEG-COHb increasing non-specific troponin I levels in sickle cell patients; however, there was no laboratory evidence of cardiac ischemia in over 200 patients who received PEG- COHb on a compassionate basis^60^. Animal studies, however, support the clinical role of PEG-COHb in instances of myocardial ischemia in rats and support its effectiveness in reducing infarct size if administered after the occlusion of the left anterior descending artery or at the time of reperfusion^46^. Sanguinate has been granted orphan drug status for treatment of sickle cell disease by the FDA^61,62^.

### *5.3 Limitations*

The wisdom from the lessons learned from the failed trials of OTAs has rekindled the interest of researchers and regulators enough to undertake further development of new agents. Among the most promising new OTA is the product ErythroMer (KaloCyte Inc., Baltimore, MD), which is in the initial stages of chemical development and animal testing. The product has a strong research focus emulating normal RBC physiologic interactions with O_2_ and NO. The design characteristics and physical properties of ErythroMer have preserved the hemodynamic and cellular interactions with the physical properties exhibiting its extended dry storage benefits signifying the implications for its portability and use^63^. Although previous clinical trials ended with a setback, the lessons learned from those studies have impacted greatly towards the optimization of the developed products^64^. Among a few products that have shown promise in the initial phase of development are Hemo2Life^Ⓡ^ (Hemarnina, Morlaix, France), a lugworm hemoglobin extract which is not packed into erythrocytes or other membranes. Hemo2life has natural superoxide-dismutase-like activity that may mitigate the oxidant burden attributable to HBOCs. The product has been studied for prolonging storage of transplant organs and improving post-transplant graft function^65^. Other investigational HBOCs currently in the initial phases of development are OxyVita^Ⓡ^ (OXYVITA Inc., New Windsor, NY), a stroma-free cross-linked bovine hemoglobin, HbVesicles (Terumo Corp., Tokyo, Japan), a liposomal-encapsulated human hemoglobin, and HemoAct (Japan Blood Products Organization, Tokyo, Japan), a human hemoglobin that is linked with human albumin molecules^66-68^.

## 6**. Conclusion**

Although there has been much interest in developing a blood substitute spanning many decades, development of a successful OTA has not been fruitful to date. This lack of success is perhaps as much attributable to toxicities related to the agents in development as it is to the physiologic nuances of O_2_ delivery to the tissues. Yet, the future does hold promise as new agents, particularly hemoglobin-based agents, are already in the pipeline and one agent has achieved FDA orphan drug status for the treatment of sickle cell disease.

## **KEY POINTS**

**◊** **Blood substitutes (“artificial blood”), better termed as oxygen therapeutic agents (OTAs), have been in development for many decades**

**◊** **The development of OTAs has taken two main approaches: 1. perfluorocarbon-based substitutes and 2. hemoglobin-based oxygen carriers**

**◊** **Currently, there are no Food and Drug Administration (FDA)-approved OTAs given the toxicities of these agents, though some OTAs are used clinically outside of the United States**

**◊** **It is possible to use OTAs in the United States via the FDA expanded (compassionate use) access program for selected patients with severe life-threatening anemia**

**◊** **There are promising new developments in the search for a safe and effective OTA**
